# Barotrauma Linked to Coronavirus Disease 2019 Infection in Younger Patients: A Case Series

**DOI:** 10.7759/cureus.14573

**Published:** 2021-04-20

**Authors:** Thomas G Ng, Eric Degaetano, Usha Trivedi, Morium Akthar

**Affiliations:** 1 Internal Medicine, Rutgers University, Newark, USA; 2 Pulmonary Critical Care Medicine, Rutgers University, Newark, USA

**Keywords:** coronavirus disease 2019 (covid-19), barotrauma, pneumomediastinum, pneumothroax, invasive mechanical ventilation, severe acute respiratory distress syndrome, rutgers new jersey medical school

## Abstract

Patients infected with coronavirus disease 2019 (COVID-19) on invasive mechanical ventilation were found to have high rates of barotrauma. Herein, we present five patients admitted to the intensive care unit between March and April 2020, who developed barotrauma as a complication of COVID-19 pneumonia. This series includes four males and one female with a mean age of 54 years, most without significant chronic comorbidities or former tobacco use. All were intubated for hypoxic respiratory failure due to the COVID-19 infection. The diagnosis of barotrauma was confirmed via radiography showing the presence of pneumothorax, pneumomediastinum, or subcutaneous emphysema on radiographic imaging. At the time, they were evaluated for convalescent plasma infusion, remdesivir, and interleukin-6 inhibitor. Each of the five patient's hospital courses were documented. The average number of days between intubation and subsequent barotrauma was 6.8 days with the mean length of hospital stay being 49 days. Three of the five patients passed away due to complications related to COVID-19. Due to the unknown nature of the virus, our findings add to the growing evidence that those infected, even without significant comorbidities, are at high risk for pulmonary complications and in-hospital mortality.

## Introduction

Coronavirus disease 2019 (COVID-19), caused by the severe acute respiratory syndrome coronavirus 2 (SARS-CoV-2) infection, is responsible for over 86 million cases and 1.8 million deaths worldwide. According to Edwards et al., 26% of those infected with the virus progress to acute respiratory distress syndrome (ARDS) requiring endotracheal tube placement and mechanical ventilation [1]. Pulmonary barotrauma is a potential complication of invasive mechanical ventilation and is a poor predictor of morbidity and mortality. As described by Ioannidis et al., it occurs as a result of excess volume exerted on lung parenchymal tissue and can cause alveolar rupture, leading to pneumothorax, subcutaneous emphysema, or pneumomediastinum [2]. 

With fatality rates as high as 13%, those infected are at high risk for poor outcomes, especially those above the age of 75 and those with significant comorbid conditions. As McGuiness et al. described, those on mechanical ventilation were found to have higher rates of barotrauma than comparative patients with ARDS and had higher than anticipated rates of mortality, especially in younger patients [3]. Previous research by Ji et al. investigated inflammatory markers as possible predictors of mortality [4]. That study trended leukocyte count, C-reactive protein (CRP), erythrocyte sedimentation rate (ESR), D-dimer, ferritin, and lactate dehydrogenase levels (LDH) of patients and concluded that elevation in those inflammatory markers were likely indicative for severe infection. 

In this case series, we observe the hospital course of five patients admitted to the intensive care unit in order to better understand the possible relationship between severe infection and poor in-hospital outcomes. 

## Case presentation

Case 1

**Figure 1:**
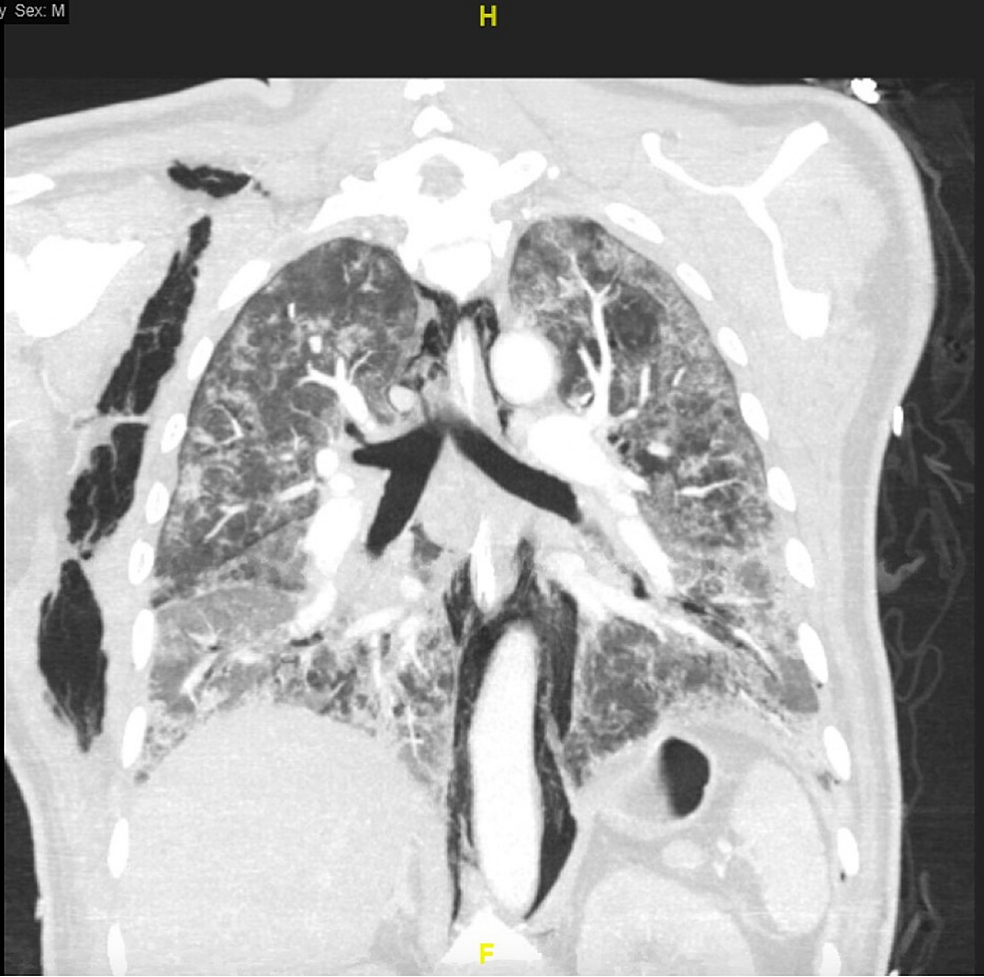
Coronal computed tomography (CT) showing extensive pneumomediastinum with air extending into the right supraclavicular region and the soft tissue of the right lateral chest wall

This 58-year-old male, with a Charlson Comorbidity Index (CCI) of 2 and a history of uncontrolled diabetes mellitus and former tobacco use, presented with two days of dyspnea. Initial laboratory results were significant for a white blood cell count (WBC) of 12.7 x 10^3^ u/L with 3.5% lymphocytes, a CRP of 396 mg/L, an ESR of 146 mm/hr, a D-dimer of > 7,800 ng/mL, a ferritin of 2,348 ng/mL, and an LDH of 615 u/L. He was initially treated with doxycycline and ceftriaxone. Convalescent plasma was given the following day. He was not a candidate for tocilizumab due to his uncontrolled diabetes mellitus. Despite treatment, the patient was intubated on Day 4 due to respiratory distress. Because of persistent hypoxemia, computed tomography (CT) was done four days post-intubation which showed supraclavicular subcutaneous emphysema, pneumomediastinum, and bilateral pulmonary emboli (PE) (Figure 1). Fortunately, the patient responded to treatment, was converted to a tracheostomy, and was discharged to acute rehabilitation after 59 days.

Case 2

**Figure 2:**
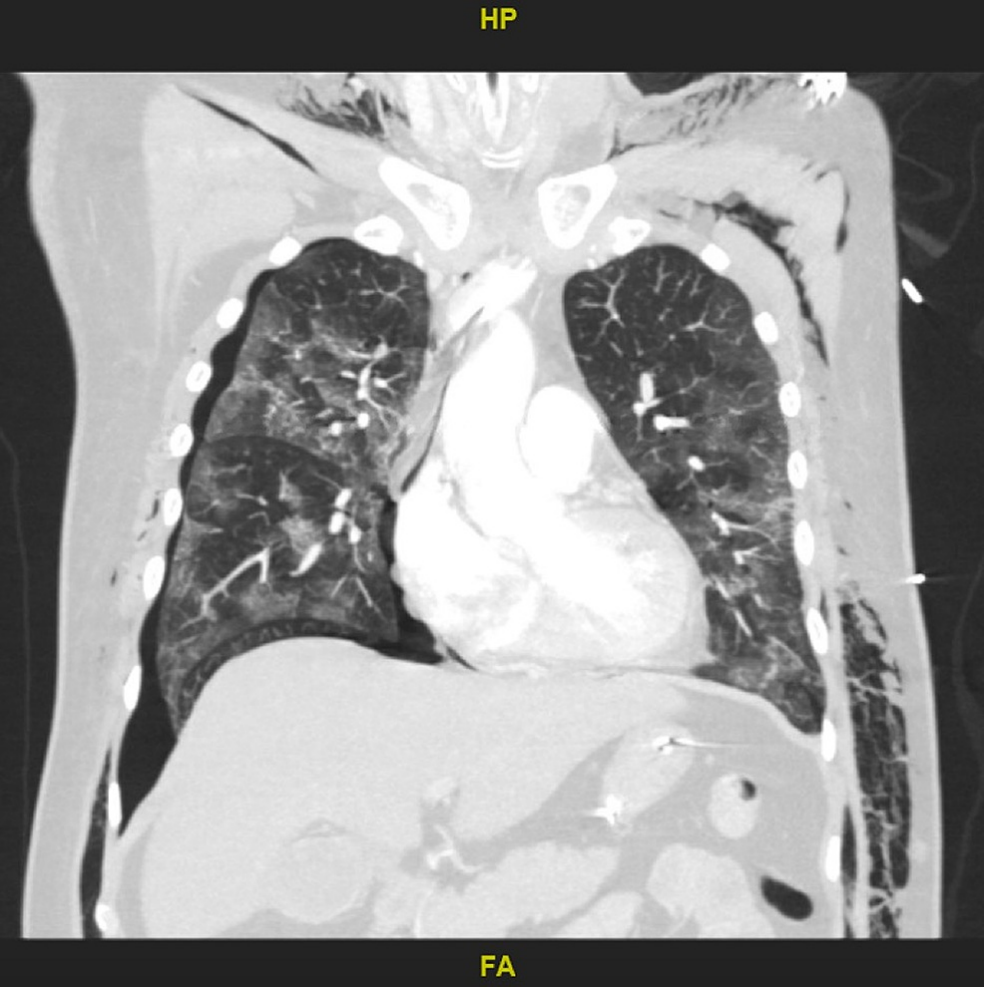
Coronal computed tomography showing extensive subcutaneous emphysema and a right-sided pneumothorax that required thoracostomy tube placement

This 49-year-old male, without a significant past medical history and a CCI of 1, was emergently intubated due to severe respiratory distress by emergency medical services prior to admission. He had a WBC count of 19.4 x 10^3^ u/L with 17.3% lymphocytes, a CRP of 331 mg/L, an ESR of 145 mm/hr, a D-dimer of > 7,800 ng/mL, ferritin of 3,247 ng/mL, and an LDH of 750 u/L. The patient was started on ceftriaxone and doxycycline and received tocilizumab on Day 2. Due to persistent hypoxemia, CT with contrast on Day 3 was done and revealed the presence of bilateral PEs, extensive subcutaneous emphysema, and a right-sided pneumothorax that required thoracostomy tube placement (Figure 2). In addition to coagulopathy, the patient also developed acute renal failure requiring emergent hemodialysis and *Acinetobacter *sepsis requiring vasopressor support. Despite intervention, he continued to deteriorate and died on Day 21 of his hospital stay. 

Case 3

**Figure 3:**
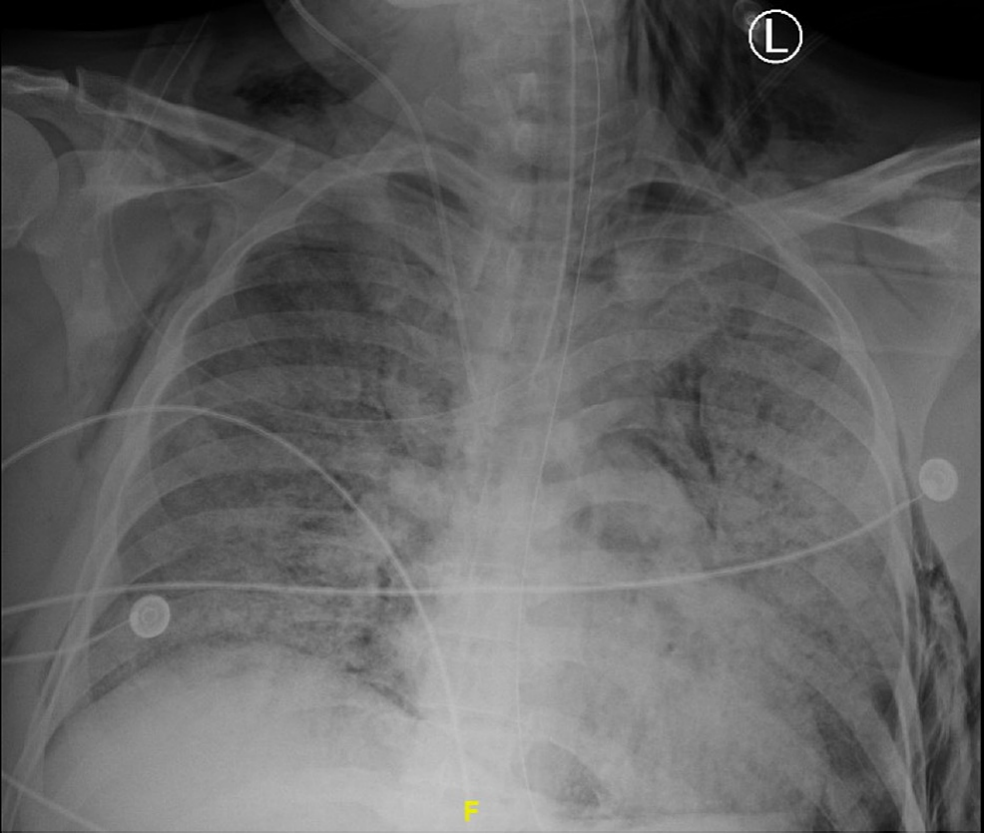
Chest radiograph showing right-sided subcutaneous emphysema and right-sided apical pneumothorax

This 39-year-old male, without a significant past medical history and a CCI of 1, presented with one week of dyspnea. Admission laboratory studies showed a WBC count of 15.1 x 10^3^ u/L with 8.8% lymphocytes, a CRP of 278 mg/L, an ESR of 98 mm/hr, a D-dimer of 6,854 ng/mL, a ferritin of 2,264 ng/mL, and a LDH of 891 u/L. He was started on doxycycline and ceftriaxone. Unfortunately, despite receiving tocilizumab, convalescent plasma, and remdesivir, the patient was emergently intubated on Day 11. Imaging 10 days post-intubation was significant for a right-sided apical pneumothorax (Figure 3). The patient’s intensive care unit (ICU) stay was further complicated by methicillin-resistant *Staphylococcus aureus *bacteremia and cardiac arrest on Day 20. He was able to be resuscitated, stabilized, underwent a tracheostomy placement after 103 days, and was discharged to an acute rehabilitation facility. 

Case 4

**Figure 4:**
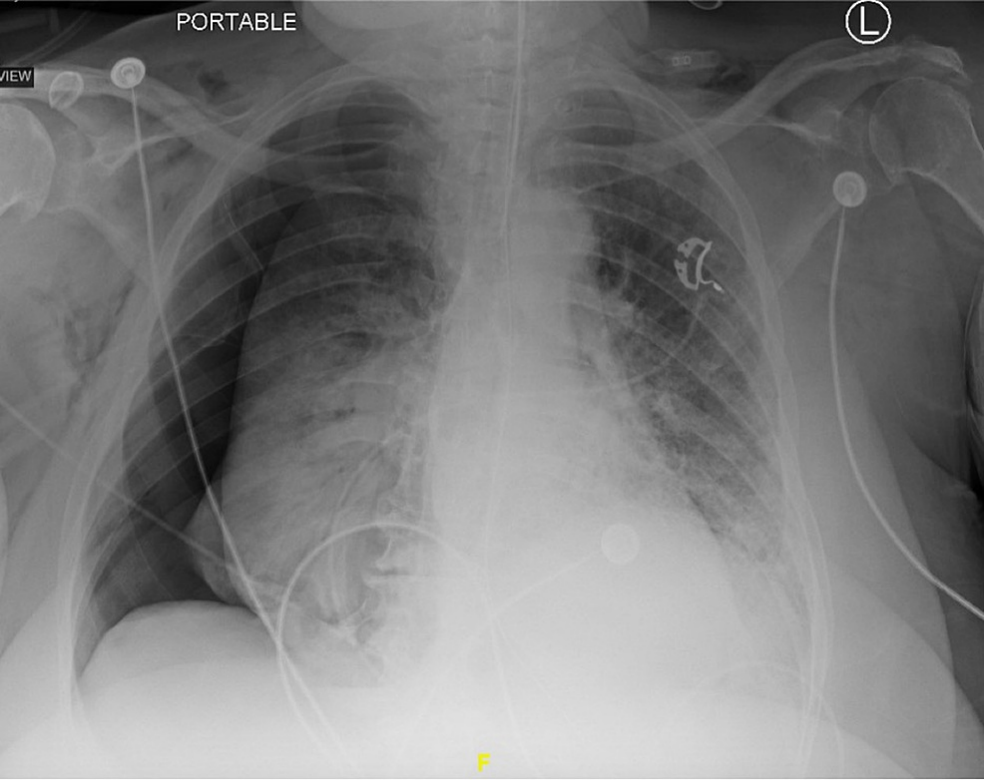
Chest radiograph of a large right-sided pneumothorax with leftward mediastinal shift requiring the placement of thoracostomy tubes

This 61-year-old female with a CCI of 3, history of hepatitis C infection, hypertension, diabetes mellitus, and former tobacco use presented with three days of dyspnea, fevers, and altered mentation. Her labs were significant for a WBC count of 13.0 x 10^3^ u/L with 10.1% lymphocytes, a CRP of 108 mg/L, an ESR of 56 mm/hr, a D-dimer of > 7,800 ng/mL, a ferritin of 1,596 ng/mL, and an LDH of 672 u/L. She was started on vancomycin and Zosyn on admission for empiric coverage, given her immunocompromised state. The patient was not a candidate for tocilizumab due to underlying hepatitis and the patient's family declined convalescent plasma and remdesivir therapy. Unfortunately, her hospital stay was complicated by cardiac arrest from hypoxemia and required emergent intubation and transfer to the ICU after achieving the return of spontaneous circulation. Follow-up imaging three days post-intubation revealed a large right-sided pneumothorax requiring emergent placement of thoracostomy tubes (Figure 4). Despite the resolution of the patient’s pneumothorax, the patient’s respiratory status continued to decline, and she died on Day 29 of her hospital stay. 

Case 5

**Figure 5:**
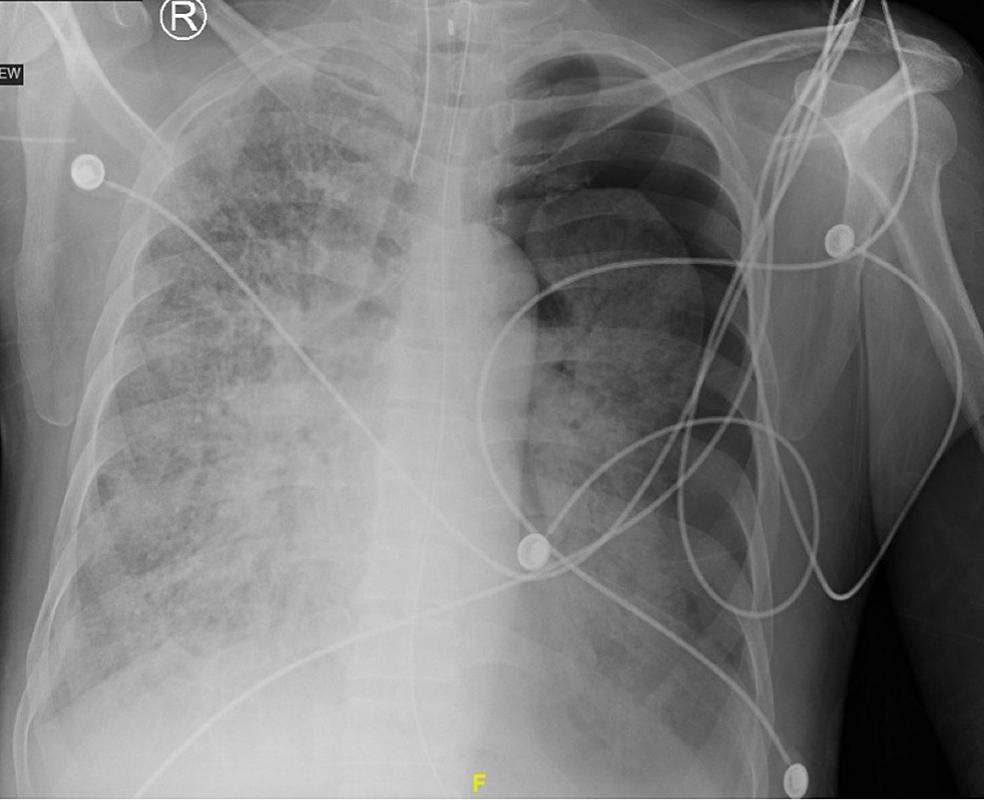
Chest radiograph showing a large left-sided apical pneumothorax with collapse of the left lung

This 63-year-old male, with a CCI of 2, presented with five days of cough, weakness, dyspnea on exertion, and fevers. Admission labs showed a WBC count of 4.5 x 10^3^ u/L with 17.1% lymphocytes, a CRP of 377 mg/L, a D-dimer of 7,338 ng/mL, a ferritin of 3,536 ng/mL, and an LDH of 831 u/L. He was started on doxycycline, ceftriaxone, and received two doses of convalescent plasma within the first week of admission. The patient initially deferred tocilizumab due to associated risk. The patient's hospital stay was complicated by lower extremity thromboses requiring intravenous anticoagulation. He was intubated on Day 8 for persistent hypoxemia despite bilevel oxygenation. Two days after intubation, thoracostomy tubes were placed to treat a large left apical pneumothorax seen on chest radiography (Figure 5). The patient's clinical course was further complicated by *Klebsiella* ventilator-associated pneumonia requiring multiple vasopressors, disseminated intravascular coagulation requiring multiple transfusions, and acute renal failure requiring hemodialysis. Despite the aggressive intervention, including the addition of IL-6 antagonist therapy later on, the patient went into asystole on Day 33.

## Discussion

According to the Center for Disease Control, recent data still shows that those patients at the highest risk of death are those above the age of 75 years and those with chronic diseases, especially cardiovascular and pulmonary comorbidities [5]. It is not entirely understood how younger patients without such comorbidities have high rates of mortality. This may be due to the rapid onset of ARDS with the COVID-19 infection, which puts patients at a high risk for complications, such as barotrauma. The average age of the five patients in this series was 54 years, with an average CCI score of 1.8, indicative of a 90% 10-year survival percentage. Three of the five patients were non-smokers and two were former smokers. All of the patients reviewed did not have a prior history of pulmonary disease. The average number of days between admission and intubation was 6.2 days and the average time from intubation to the diagnosis of barotrauma was 6.8 days. Four out of the five patients required thoracostomy tubes due to cardiopulmonary compromise. The total hospital length of stay for these patients ranged from 21 days to 103 days, with an average of 49 days. 

Some studies have speculated the role of inflammatory markers as possible predictors of poor outcomes. Kolani et al. suggested that an elevated cytokine storm leads to the compromise and rupture of the alveolar membrane [6]. The average ferritin and D-dimer levels of the three that suffered inpatient mortality was 7,800 ng/mL and 2,793 ng/mL, respectively, compared to the average levels of the other two patients which were 7,327 ng/mL and 2,306 ng/mL, respectively (Table 1). However, a larger sample size will be needed in order to study general trends and the likelihood of complications. Another possible predictor of poor prognosis was the severity of lymphopenia, especially those with lymphocyte counts of less than 1,100 u/L. Of the three with in-hospital mortality, one had severe leukopenia. The combination of inflammatory markers and leukopenia may be useful in determining risk of complications in younger or otherwise healthy patients. 

**Table 1:** Patient Inflammatory Markers on Admission CRP: C-reactive protein; ESR: erythrocyte sedimentation rate; LDH: lactate dehydrogenase

Case	CRP (mg/L)	ESR (mm/hr)	D-Dimer (ng/mL)	Ferritin (ng/mL)	LDH (u/L)
1	396	146	> 7,800	2,348	615
2	331	145	> 7,800	3,247	750
3	278	98	6,854	2,264	891
4	108	56	> 7,800	1,596	672
5	377	-	7,400	3,536	831
Average	298	-	-	2,598	751

## Conclusions

The presence of barotrauma in those infected with COVID-19 is possibly a negative prognostic indicator for poor outcomes. Our findings emphasize how this viral pneumonia poses a threat to younger patients without significant comorbidities and those infected are at high risk for in-hospital mortality. The observations from this series also contribute to the growing knowledge of the disease’s pathophysiology and progression in order to aid in better management of this potentially fatal illness. 
